# Is nutrition science ready for the twenty-first century? Moving towards transdisciplinary impacts in a changing world

**DOI:** 10.1007/s00394-020-02241-0

**Published:** 2020-04-29

**Authors:** Adèle R. Tufford, Philip C. Calder, Pieter Van’t Veer, Edith F. Feskens, Theo Ockhuizen, Aletta D. Kraneveld, Jan Sikkema, Jan de Vries

**Affiliations:** 1 Division of Human Nutrition and Health, Wageningen University and Research, Wageningen, The Netherlands; 2 Faculty of Medicine, University of Southampton, Southampton, UK; 3 NIHR Southampton Biomedical Research Centre, University Hospital Southampton NHS Foundation Trust and University of Southampton, Southampton, UK; 4 Nutricom Consultancy, Rumpt, The Netherlands; 5 Division of Pharmacology, Utrecht Institute for Pharmaceutical Sciences, Faculty of Science, Future Food Utrecht, Utrecht University, Utrecht, The Netherlands; 6 Center for Development and Innovation, University Medical Center Groningen, Groningen, The Netherlands; 7 De Vries Nutrition Solutions, Gorssel, The Netherlands; 8 Foundation Nutrition in Transition, Gorssel, The Netherlands

## Abstract

*Malnutrition in an obese world* was the fitting title of the 13th Federation of European Nutrition Societies (FENS) conference held in October 2019. Many individuals do not eat a healthy, well-balanced diet, and this is now understood to be a major driver of increased disease risk and illness. Moreover, both our current eating patterns and the food system as a whole are environmentally unsustainable, threatening the planetary systems we depend on for survival. As we attempt to feed a growing global population, food systems will increasingly be confronted with their environmental impacts, with the added challenge of climate change-induced threats to food production. As we move into the third decade of the twenty-first century, these challenges demand that the nutrition research community reconsider its scope, concepts, methods, and societal role. At a pre-meeting workshop held at the FENS conference, over 70 researchers active in the field explored ways to advance the discipline’s capacity to address cross-cutting issues of personal, public and planetary health. Using the world cafe method, four themed discussion tables explored (a) the breadth of scientific domains needed to meet the current challenges, (b) the nature and definition of the shifting concepts in nutrition sciences, (c) the next-generation methods required and (d) communication and organisational challenges and opportunities. As a follow-up to earlier work [1], here we report the highlights of the discussions, and propose the next steps to advance responsible research and innovation in the domain of nutritional science.

## Introduction

The twenty-first century challenges faced by nutrition scientists are immense: a “triple burden of malnutrition”, namely overnutrition and obesity, undernutrition and nutritional deficiencies [2–4]; unsustainable food supply chains; policy inertia and distrustful consumers. In the context of globally limited resources and social disparities, it would be a gross oversimplification to say that increased production is sufficient to achieve food security, and that better dietary choices would alleviate obesity, undernutrition and non-communicable diseases (NCDs). Combined with the impacts of climate change on both the nutritional content and supply of foods [4–7], this requires that we re-think how nutrition research is performed. How will we provide dietary guidelines against this backdrop of a changing food system, while regaining the trust of consumers and citizens? What types of research avenues are needed to design, test and supply the healthy and sustainable diets of the twenty-first century?

A recent assessment by the Dutch coalition Nutrition in Transition (NiT) explored some of these current challenges facing nutrition sciences and concluded that maintaining the field’s capability will require the incorporation of new methodologies to answer the complex and challenging twenty-first-century problems related to food, nutrition and health [1]. At the same time, the credibility of nutrition science in the eyes of both policymakers and consumers requires revised organisational and financial structures to both carry-out and clearly communicate relevant research. NiT, the Food Nutrition and Health Research Infrastructure (FNH-RI) and the Federation of European Nutrition Societies (FENS) share a common goal of consensus and community building, co-creation, foresight generation and innovation within the nutrition sciences (Box 1).

**Box 1:** Overview of the participating groups: a shared mission to ensure nutrition science is fit for the twenty-first century

***Nutrition in Transition (NiT):*** A coalition of Dutch Nutrition Scientists seeking to facilitate and shape discussions on the future of nutrition sciences via publications and communications, workshops and conferences.
***Food, Nutrition and Health Research Infrastructure (FNH-RI):*** A pan-European initiative to design and implement a distributed research infrastructure that facilitates research on sustainable diets for twenty-first-century citizens. FNH-RI will unite fragmented research fields on sustainable food supply, food environments, consumers’ eating patterns and personal and public health outcomes, by connecting research-, industry-and citizen-generated data and facilities.
***Federation of European Nutrition Societies (FENS) governance and working groups:*** three FENS working groups have been recently established, concerning (1) the concepts and methodologies required for credible nutrition science, (2) organisation, capabilities and funding structures, and (3) the communication of nutrition science to the public, patients, medical community and industry [8].

**Fig. 1:**
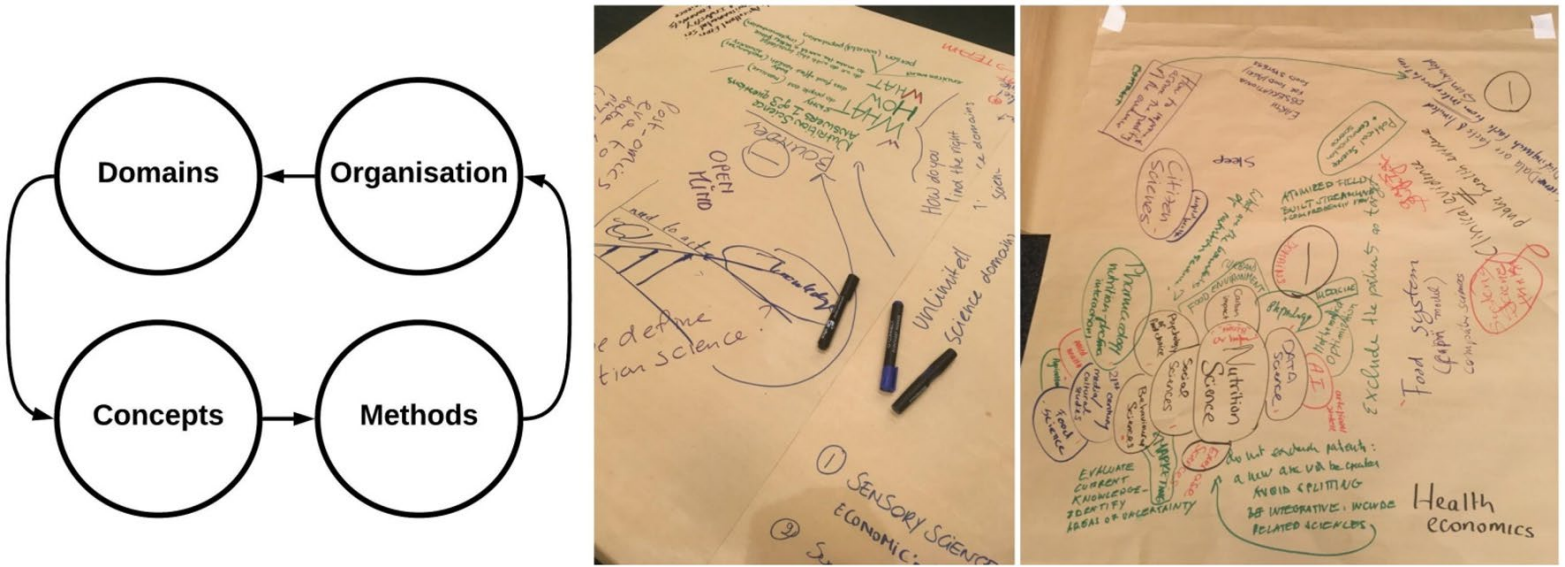
The World Cafe method: examples of collaborative discussion output. The workshop principles followed the World Café method, using the following principles: set an appropriate context; create a hospitable space; explore questions that matter; encourage everyone’s contribution; connect diverse perspectives; listen together for patterns and insights; and sharing of collective discoveries [42]. Each participant followed an individualised route around the tables to ensure interaction with a different group of individuals at each table visited

Putting these goals into action, a satellite workshop was held prior to the October 2019 FENS conference in Dublin, attended by representatives from NiT, FNH-RI and FENS, in addition to 60 additional researchers active and engaged in the field. The workshop used the ‘World Cafe’ method (Fig. 1) of guided, facilitated interactive discussions, broadly related to advancing the discipline’s capacity to address pressing personal and planetary health issues while regaining public trust. The four themes explored in depth were: the domains critical for the research of food, nutrition and health, and their societal contexts; concepts in nutrition sciences; advanced methods in nutrition sciences; and community building and organisation within the field. The exploration of these themes defined the transdisciplinary research and dissemination needed to produce healthy, sustainable, acceptable, safe and accessible diets for all. Such ambitious goals need to be driven by a reinvigorated and united scientific community, in tune with both policy and public concerns. The time for initiating this much needed scientific-societal co-creation is now; acting rapidly and decisively will allow the field to capture the momentum from reports and landmark commissions on food, health and sustainability such as the EAT-Lancet Commission [9], the High-Level Panel of Experts and Guidelines for Sustainable Healthy Diets (FAO-WHO) [10], the European Public Health Association Report on Healthy and Sustainable Diets for European Countries [11], the Rockefeller Foundation-Lancet Commission on Planetary Health [12] and the Lancet Global Syndemic [4]. The following is a capitulation and discussion of the main findings that arose from the workshop, under the four broad themes that were explored. These outcomes have provided critical input into several ongoing initiatives, including the FENS working group ‘Concepts and Methods in Nutrition Sciences’ [8] and priority-setting and progress towards the pan-European FNH-RI.

## Building bridges in nutrition sciences

Human nutrition is currently dominated by a pernicious food supply chain and obesogenic food environments, dependent on threatened planetary systems that have already crossed the boundaries for resilient functioning. Broad transitions in the nutrition sciences have historically mirrored times of sweeping change in food landscapes; for example, during food shortages or nutrition-related epidemics [13, 14]. The tremendous pressure to transition to a healthy and sustainable food system means the discipline of nutrition sciences must adapt or perish. Designing the healthy and sustainable food production and supply chains, food environments, and diets that are needed to support a growing population will require a convergence of disciplines, and a fostering of systems-level rather than individual-level thinking [15].

**Fig. 2:**
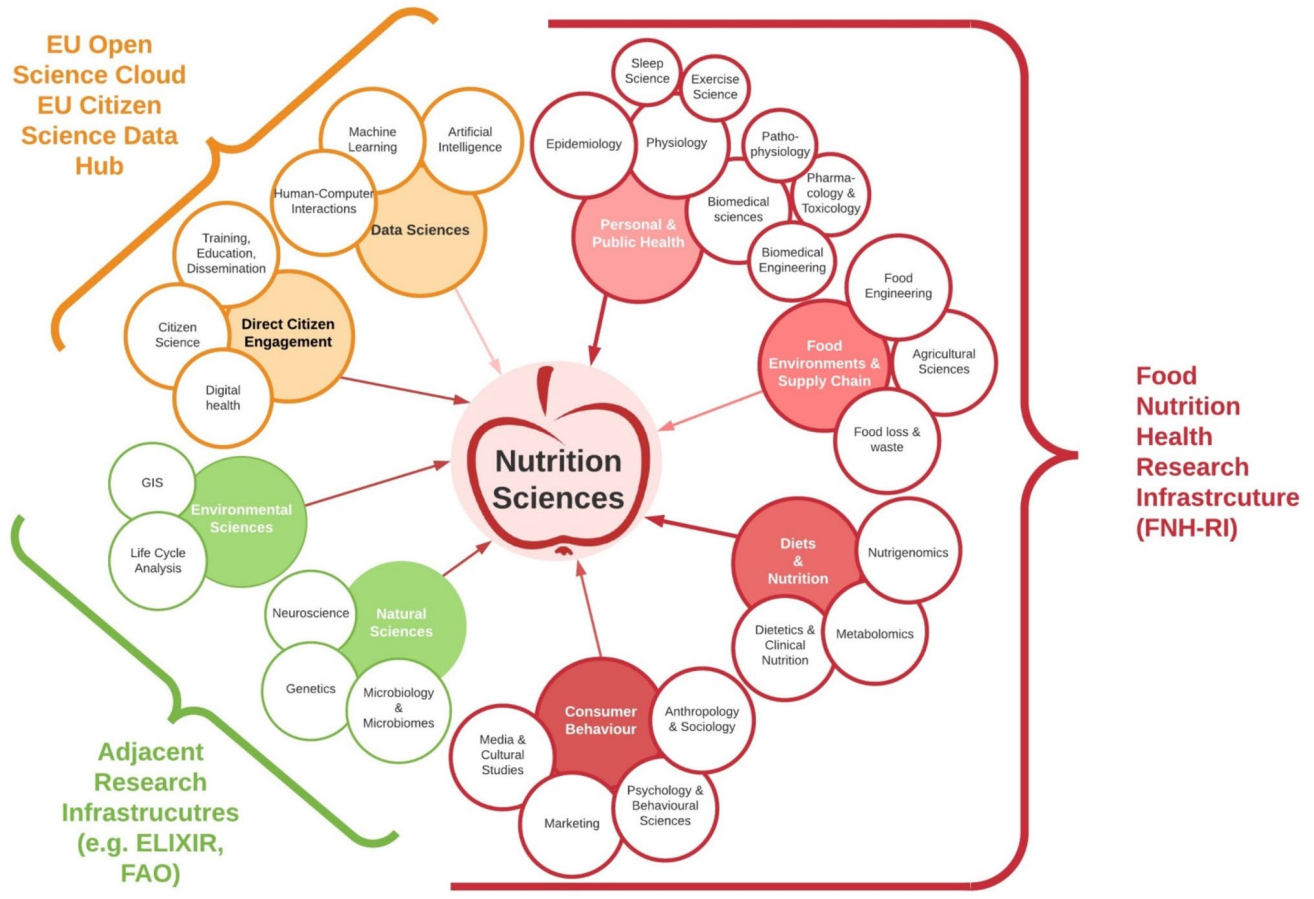
What is Nutrition Science? An adapted cloud chart of the domains and disciplines identified as critical to the future of nutrition sciences in collaborative workshop discussion. The overarching bodies with the capacity to cross-fertilise and integrate the data, methods and training between disciplines are noted in brackets, with the relevant disciplines they currently cover, or propose to cover in the near future. FNH-RI and the EU Open Science Cloud are infrastructures that are still in planning or have not reached maturity

At the 2019 pre-FENS workshop, the domains table asked: what domains of science are necessary to address key issues related to food, nutrition and health? The domains identified were both numerous and broad in scope (Fig. 2): ranging from molecular biology to social media studies. The need to link the personal determinants of health (which itself can range from genes to social environment) to food environments was highlighted as a critical step towards elevating nutrition sciences to systems-level thinking, leading to systems-level impacts. The domains noted spanned ranges of both biological complexity and time scales. On an individual level, the domains encompassed e.g. short-term metabolic effects of nutrients during a single day vs. studies of nutrition across a lifetime (in utero vs. childhood vs. the elderly). On a systems level, the domains encompassed e.g. studies of the impact of consumer food choices on short-term supply and demand feedback loops vs. their impact on future generations of humans inhabiting the planet, and the biophysical processes supporting life.

Eight major domains were identified by workshop participants: Personal and Public Health, Food Environments and Supply Chain, Diets and Nutrition, Consumer Behaviour, Natural Sciences, Environmental Sciences, Direct Citizen Engagement, and Data Sciences (Fig. 2). These broad domains were then further divided into sub-disciplines, such as Nutrigenomics, Metabolomics and Dietetics and Clinical Nutrition, under the ‘Diets and Nutrition’ domain. Simply naming a breadth of domains, however, is insufficient to ensure that insights from other fields are integrated into studies concerning nutrition. Linking disciplines to allow truly transdisciplinary research may require the creation of entirely new entities dedicated to building bridges between fields; whether those bridges be digital (e.g. data standards and interoperability) or physical (e.g. sharing and dissemination of advanced methods or labs, facilities and tools traditionally confined to particular disciplines). We must also consider practical approaches to bridge the deep divide between social sciences and life sciences-centred approaches to food and nutrition, to arrive at systems-level insights. Among the eight domains deemed critical for the future of nutrition science, FNH-RI is in the process of building a distributed (i.e. predominantly virtual) pan-European research infrastructure consortium, which will link four critical domains, namely Personal and Public Health, Food Environments and Supply Chains, Diets and Nutrition and Consumer Behaviour. Via a hub of data science experts, FNH-RI will link existing and emerging data in these fields with advanced facilities and tools – including a pan-European consumer data platform to allow advanced modelling of consumer behaviour. It will also provide the training and education required to functionally link domains, as well as perform advanced data science and nutritional assessment methods. By allowing interoperability of meta-data and expertise present in already-existing consortia, e.g. EiTFood, METROFOOD and JPI-HDHL, FNH-RI will provide a cutting-edge infrastructure allowing transdisciplinary research on the biological and social determinants of food choice. The domains of the FNH-RI have been coined ‘from farm-gate to hospital door’; thus while it addresses interoperability in many critical domains relevant to nutrition science, due to practical constraints, it has chosen to focus on diets from the consumer perspective.

How can nutrition sciences crosstalk more effectively with domains traditionally considered ‘outside of its realm’? Environmental survey and agricultural data has the capacity to inform the nutritional content and planetary impact of foods at considerably greater granular detail [16, 17]. Linking to these aspects of food could be accomplished by stronger interoperability with, e.g. agricultural and environmental monitoring research infrastructures and platforms such as EO4Agri [18] and the FAO databases (CountrySTAT [19] and INFOODS [20]), as well as initiatives such as Global Open Data for Agriculture and Nutrition (GODAN) [21]. In the natural and medical sciences where researchers are often working on genes or diseases without direct links to nutrition critical insights, resources or data with pertinence to medical nutrition, nutrigenomics and metabolism can go unnoticed when disciplines remain siloed. Likewise, other medical sciences have much to gain from considering nutrition for example, the exciting new frontier of science exploring the link between microbiota and brain functioning [22]. Creating stronger and more interoperable links between nutrition sciences and large research consortia such as ELIXIR [23] (genetics, chemical/molecular biology and metabolism), ECRIN [24] (multi-national clinical trials) and EATRIS [25] (translation of discovery to medical products) can only be mutually beneficial.

Underpinning advances in interoperability across domains requires a full embrace of data science and advanced information technology capacities not only through institutional channels such as the European Open Science Cloud (EOSC) [26], but also by exploiting what big tech industry has available to share, from the deep capacities of Google Maps to inform our understanding of food environments, to emerging trans-dermal sensors of caloric intake. These new data sources expand even further with Citizen Science and the vast amounts of personal data available outside of the traditional epidemiological research cohort studies, such as e.g. purchase data and smart gadgets. Nutrition scientists are tasked with keeping pace with *both* what citizens are personally reporting and demanding of their food environment, as well as providing the means to perform sound science needed to inform these choices and demands, as well as aligning to the European Data Protection Board and the General Data Protection Regulation (GDPR) to respect consumer and patient privacy. The EU Citizen Science and Smart Cities Data Hub [27] is one start to engaging with the wide range of emerging initiatives. However, researchers may also find it more accessible or valuable to tune into community or topical efforts in a more participatory way; after-all, in addition to researchers, we are citizens, consumers and data providers ourselves.

‘Soil to soil’ captures the domains needed for a healthy and sustainable food system and supply of nutrients, supplied by supportive food environments, resulting in optimal personal and planetary health. Nutrition scientists have many options, especially emerging over the next decade, to operate in conjunction with other fields.

## Changing nutrition concepts in a changing world

Before branching and expanding the domains within which nutrition sciences operate, the community must arguably first reach a consensus on what we wish to measure and what we wish to achieve. What does it mean to study ‘Health’, to obtain ‘Evidence’ or ‘Proof’ or to determine ‘Causality’? Achieving consensus on these concepts is an important precursor to the building of standards, ontologies and vocabularies that make our findings interoperable with many of the other domains described above. At the concepts table, workshop participants were asked to reconsider concepts such as ‘health’, ‘proof’ and ‘causality’ to work towards a shared ontology for nutrition sciences.

### Health: scope and definitions

Health is something we all seek to improve. But is health within one human body, that of an entire community or group, or that of the planet in recognition that human health cannot be achieved without the ecological systems that support life? There is growing agreement that the standard 1948 WHO definition of health as ‘a state of complete well-being, not merely absence of disease’ [28] does not capture full human well-being nor the planetary perspective. Moreover, medical definitions of health, formulated in response the rise of NCDs in the latter half of the twentieth century, often fail to take into account what a patient or individual may consider to be most critical for their well-being [29]; this is also encompassed in the WHO’s 1986 Ottawa Charter that moves from a definition of health towards a concept - that health is a means to live, not the objective of life itself [30, 31]. Considering the central role of diets, and the food systems underlying them, in health, the FAO has also spent considerable effort to advance the concept of a healthy and sustainable diet and food system, arriving at the following explanation:

Sustainable Diets are those diets with low environmental impacts which contribute to food and nutrition security and to a healthy life for present and future generations. Sustainable diets are protective and respectful of biodiversity and ecosystems, culturally acceptable, accessible, economically fair and affordable; nutritionally adequate, safe and healthy; while optimizing natural and human resources

At the *concepts* table, this more holistic concept of health was championed for the nutrition sciences. An extension to Maslow’s classical hierarchy of socio-psychological needs culminating in ‘self-actualisation’ was proposed for health, where sound and healthy planetary systems supporting life sits at the base, followed by public and personal health - defined in the WHO sense by the absence of disease - and culminating in a state beyond well-being: thriving. Our end goal should thus be beyond planetary, public and personal health and well-being for all, and towards thriving, adaptability and capacity for resilience of both humans and planetary systems in the face of unforeseen stressors and challenges.

What does this mean for nutrition sciences? As outlined in the breadth of domains described above, a stable supply of nutritionally adequate foods must come from a healthy food environment (healthy communities) and a healthy planet (healthy and sustainable food production systems and supply chains). Significantly greater efforts should be made to include, for example, environmental or social impact assessments of proposed nutritional interventions and system changes/transitions. New foods rich in macro and micro-nutrients could be produced with a huge carbon footprint using materials sourced from around the globe, and/or produced using cheap labour in conditions unacceptable to European workers, undermining the health of individuals and communities in the developing world. As a specific example, trans fatty acids have been slowly replaced in recent decades by palm oil, which is a primary driver of deforestation and water and soil pollution in some of the world’s most ecologically sensitive regions [32, 33]. All researchers concerned with health improvement must make these considerations, particularly in the field of nutrition, which piggybacks on the nearly 25% of greenhouse gas emissions produced by the food sector. Nutrition scientists bear the responsibility of acting in line with the Sustainable Development Goals related to health and food, recognising that improving the health and nutrition of a few, at the expense of planetary or personal health of individuals in the developing world, will not benefit health overall.

### Evidence and claims of causality in the twenty-first century ‘post-truth era’

In the face of an ever-growing body of global research, combined with self-anointed experts on social media emerging as major voices in the health and nutrition realm, it is important to consider the interrelated concepts of evidence, proof and causality. While a behaviour as fundamental to our livelihoods as eating has forever been subject to personal and cultural claims of causality (and in many cases, rightfully so), today’s convergence of corporate-interest-driven unhealthy (while for most in Europe, imminently accessible) food environments and nearly unlimited information availability means that sound evidence-based nutrition science findings need to be carefully constructed and disseminated.

The concepts workshop table further explored some possible themes to consider strengthening the community’s capacity to respond to unique twenty-first-century research challenges. At the very least, base-level data quality should be improved to enable researchers to (a) better separate facts from inference, in order to inform causality (to be discussed below), and (b) allow for evidence-based rather than eminence-based driven policies and research questions. At the same time, our standards of evidence must incorporate greater acceptance of the fluidity of knowledge and the spectrum of certainty; biology and human behaviour never were, and never will be binary. Thus, greater space must be afforded to spectrums of certainty and ensuing ranges of outcomes.

Despite the acknowledgement of ‘proof’ lying on a spectrum, as scientists we must still strive for a measure of causality to advance our own research areas, lest we spin our wheels for decades trying to solve narrow problems. Moreover, with a clear concept of causality, we can make results more interoperable, translating results from one context to another – making a contribution towards the goal of better alignment between the broad research domains described earlier. A key challenge noted at the workshop was the need to extract generalised causality from dynamic systems models, fostering an improved ability to move beyond reductionist single-factor causation models to systems-level multi-factorial, dynamic models. This includes the complex adaptive systems models recently proposed by Schill et al. [15]. Casting a wide net of evidence to weigh, however, can mean that one never arrives at a fixed point of causality. How can researchers handle this shifting terrain? A particularly useful framework for advancing classical concepts of causality has come from the field of epidemiology, where Bradford Hill’s nine mid-twentieth century criteria for causality have been re-framed to incorporate new insights into e.g. complex biological systems ranging from the genetic or epigenetic effects of molecules to the role of the built environment in both health outcomes and risk-exposure [34]. Incorporating advanced data science, such as Bayesian association networks and directed acyclic graphical analyses, into causal models will lead to formalised models of inference that can be used as tools in multiple areas of nutrition science.

As a step in this direction, nutrition sciences can exploit the improved data interoperability offered by emerging platforms outlined above (e.g. the FNH-RI) and better incorporate advanced data sciences such as machine learning to perform multi-factorial and dynamic analyses. These advanced methods are elaborated further below.

## Advancing and incorporating methods to move nutrition science forwards

Expanding the scope and content of nutritional sciences via inclusion and reach to more domains, alongside the re-thinking of relevant concepts to address multi-factorial societal issues pertaining to nutrition (i.e. the Global Syndemic of undernutrition, obesity and climate change [4]), demands a controlled and validated expansion of methods used. Furthermore, scientific research is inherently ever-evolving, with continuously shifting paradigms. This implicates a continued need for the adaptation of methodologies and the ensuing outputs of these methodologies. At the pre-FENS workshop, the methods table was asked to discuss ways in which nutrition scientists could extend classic research methods towards innovative approaches addressing key societal challenges. The workshop table agreed, as was also raised by Penders et al*.* in 2017 [1], that classical methods in the nutrition sciences are insufficient to solve societal and systemic issues related to nutrition. Methods such as the randomised controlled trial and the 24-h dietary recall need a twenty-first-century upgrade. They must incorporate both the technological advances now at our disposal, and the widely accepted realisation that many of the ‘wicked’ issues in nutrition, namely the triple burden of malnutrition and its related NCDs, unsustainable food supply chains, policy inertia and distrustful consumers, have complex and multi-layered drivers [4].

Moving from single factor causality to a dynamic understanding of systems is the grand research methods challenge. First and foremost, improved data collection and quality must make use of data science and statistical methods to make data both open and FAIR (findable, accessible, interoperable and reusable), effectively managing research output so as to maximise impact [35]. From this, standards, definitions, vocabularies and ontologies emerge, which must be accompanied by more rigorous oversight and streamlined standards on the part of journals. This should also be accompanied by more coherent and uniform standards across journals for data management and presentation, as the variety of standards and practices often hinder the comparison of data on similar topics from different journals. This structure provides the scaffolding within which data is housed, allowing interoperability to both other areas of natural science and with the social sciences - for example by combining genomics and metabolomics with behaviour. This could include Bayesian network analyses to estimate the wide-spread effects of dietary interventions on NCD prevalence and outcomes, and the associated behavioural and social factors influencing the efficacy of dietary interventions. This could also resemble in silico agent-based modelling of nutritional interventions prior to roll-out, allowing the accounting for multiple levels of food system complexity and the enormous number of variables determining compliance and outcomes in vivo*.* Thus, traditional methods, i.e. the RCT, can strengthen and evolve with the aid of data science to arrive at more complex feedback loops capable of informing long term health and sustainability forecasting. At the root of these desired advances are shared ontologies and vocabularies between classical nutrition science datasets and behavioural, environmental or other qualitative food-related data, allowing next-generation data science methods to effectively cross-link disciplines, in order to arrive at truly cutting edge and intersectional foresight.

Incorporating these advanced methods requires redesigned training for the next generation of nutrition scientists. This includes, but is not limited to, strengthened statistical and data science capacities to deal effectively with datasets growing in size and complexity, and those that move from the level of single causal relationships to complex adaptive systems. For example, nutrition data arising from cohorts and interventions have not only become ‘big’, but also ‘thick’ - meaning it may not only include metrics, -omics and behavioural data, but also image-based or qualitative data on e.g. human emotions surrounding food or choice attributes. This goes hand-in-hand with the emerging understanding that broadly effective and maximally impactful nutritional interventions may not wholly succeed without consideration of the multitude of factors governing the behaviour of citizens and consumers. The importance of appropriately engaging (and ethically exploiting) certain consumer characteristics becomes all the more urgent given the pressing timeline of the climate crisis and impending food insecurity, both of which Europe is far from exempt [36]. The technical skills required to integrate and analyse these mixed qualitative and quantitative and social and life sciences datasets need to be offered to the next generation of nutrition researchers and vice-versa: data and computational scientists and stewards require discipline-specific training to make nutrition data FAIR. This could be initiated via courses on, for example, ‘Data sciences in Nutrition’, offered via pan-European training networks aligned with Erasmus and Marie-Curie and/or the upcoming FNH-RI training and education initiatives.

There is an increasing realisation that the involvement of citizens (so-called Citizen Science) is critical for delivering effective research of particular importance for a behaviour so firmly situated at the intersection of personal and public as eating. As incorporating Citizen Science poses a challenge to controlled data acquisition, the next generation of researchers require the medium and means to redesign scientific methods to exploit citizen science, *n* = 1 studies and the big data on eating that can be deduced from online retail and social media in order to e.g. deduce the precise role of the food environment in food choice. Finally, in practical terms, the methods table explored the issue that if indeed the next generation of nutrition scientists requires advanced and extended training, it follows that certain topics and methods may then need to be excluded from the classical nutrition curriculum. Recognising that training time and capacities are limited, what gets taken out? What dated knowledge no longer serves the next generation? Societies such as IUNS, FENS and individual universities/academic institutions, in collaboration with young researchers, need to begin these important dialogues.

## Communication and trust

Rising to meet large societal challenges while also regaining public trust requires improved communication by nutrition scientists: both to policy makers setting the research funding and public intervention agendas, and to consumers making day-to-day food choices. This communication is currently not streamlined, and is inadequate, inconsistent or conflicting, leaving consumers confused and distrustful [1, 37–39]. At the same time, engaging consumers to make healthy and sustainable food choices must move forward with the knowledge that it is neither fair nor feasible to burden individuals with the responsibility of enacting the food systems transformation themselves. Hence the need for effective, inclusive and equitable policy instruments. The final workshop table concerning communication and organisation was asked to discuss the scientific-societal co-creation pathways needed to achieve societal benefit and restore public trust. Building on the discussion surrounding the concepts of evidence and causality, how do we communicate the spectra of certainty inherent in biology to a public conditioned to digest facts as a binary true or false? To butter or not to butter? Five servings of fruits or four?

**Fig. 3:**
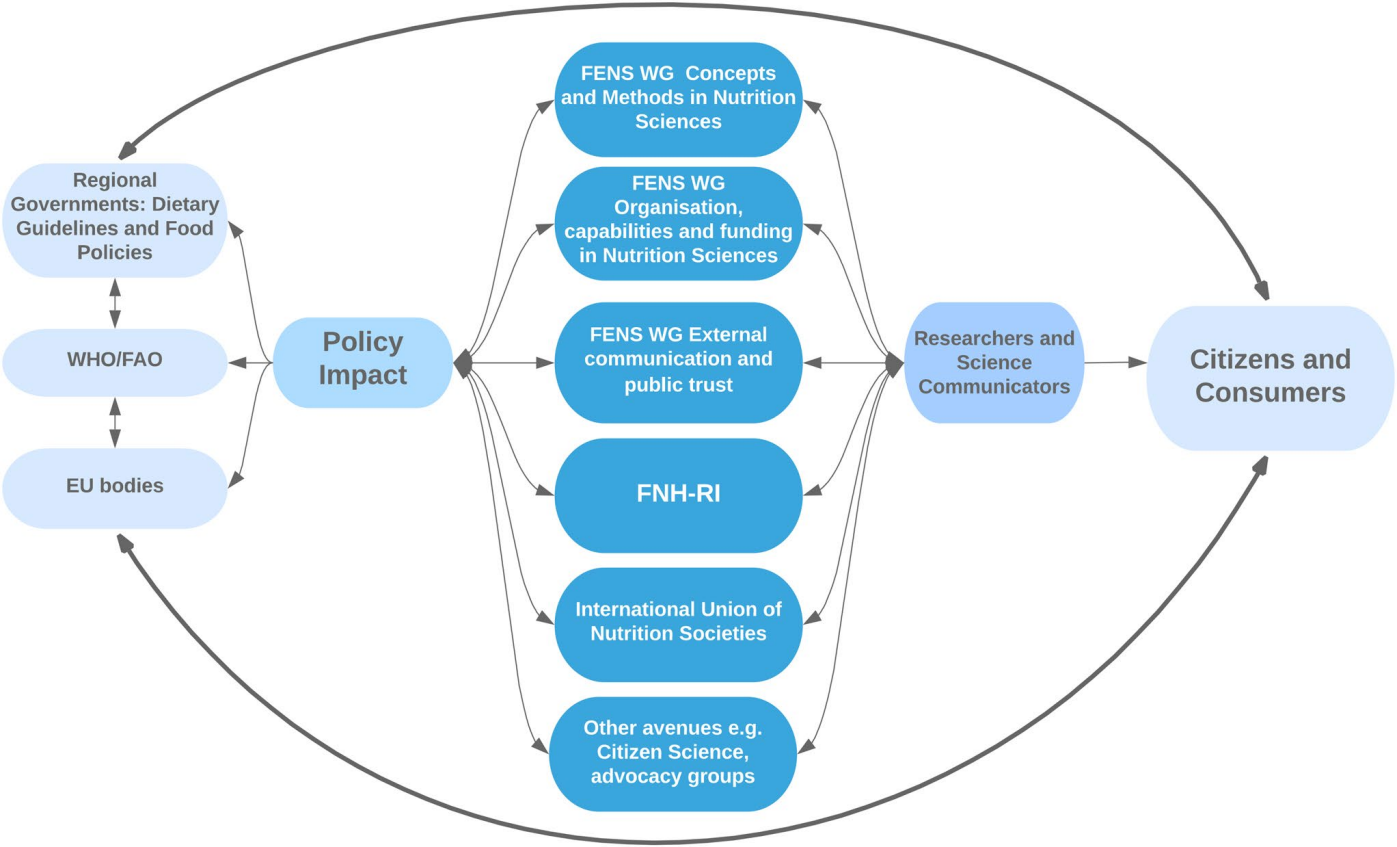
Communication and Impact. Streamlining nutrition science communication upstream to policy makers and health authorities, and downstream to citizens/consumers requires working groups and entities with targeted communication channels in a circular feedback loop. This flow chart illustrates the pathways via which redesigned organisational communication structures could lead to societal impacts

A starting point is to create specific working groups for improved upstream and downstream communication (Fig. 3). The working groups resulting from FENS 2019 are described in Calder et al. 2020 [8]. To this end, FENS is enacting a working group on external communication and public trust, which aims to, among other goals, build a united strategy in the upcoming years to unify communication to policymakers as well as to science journalists and communicators. Guidelines and strategies for communicating uncertainties, spectra of relevance and impact and the fluid nature of what is fact will be developed. This will coach researchers to move beyond communicating the results of single studies in isolation and towards conveyance of the totality of the evidence. Moreover, mechanisms to train established and new researchers on scientific communication either with the public, in general debate, or among researchers of other fields and disciplines, will be explored. The diversity of contexts and communication practices across Europe must also be taken into account; identifying and exploiting these to create context- and location-specific communication strategies will maximize research impact and social engagement in the food systems transformation.

## Growing as a community and conquering the table issues

A multitude of additional issues continues to challenge the nutrition science research community, from perverse incentives in reward and grant systems to conflicts of interest with the food and pharmaceutical industries. Researchers are currently constrained in their ability to think across domains (and are moreover not trained to do so), making it difficult to incorporate high-risk or transdisciplinary methods due to rigid and risk-averse grant structures. The above-described communication needs, therefore, require underpinning by improved organisational structures. To begin to organise around and tackle these issues, FENS will create working groups on concepts and methods, in addition to the organisation and communication working group described above, to begin to tackle the issues raised by the nutrition domains, concepts and methods tables. In close collaboration with FENS, this directed expertise within the research community can pave the way for FNH-RI to develop and make accessible the instruments, tools and training that enable new research avenues.

Integrating and functionally interoperating with other domains will be greatly facilitated by the emergence of the FNH-RI as a community platform and virtual space. This will allow the formation of functional connections between the required data, facilities and training of adjacent fields. Steps can be made towards the broader inclusion of transdisciplinary research ideas by access to both the medium and means for interoperable data, standards and ontologies as pertaining to food environments, consumer behaviour, diets and health. The FENS working group on concepts and methods in nutrition sciences can also begin to functionally address some of the existential issues facing researchers working at the intersection of science and society - as nutrition scientists do - such as navigating public, private and policy spheres, or the balance between fundamental and applied research avenues. This working group can interact closely with the FNH-RI and its data science capabilities; as well as with other research infrastructures and platforms such as METROFOOD, the Food Nutrition Security Cloud and Quisper; and pan-European training platforms and journals. The working group will create guidelines and standards for the use and incorporation of advanced nutrition science methods [8]. The FENS working group on organisation, capabilities and funding will aim to optimise the organisation of nutrition research by identifying key structures, capabilities and interactions within the community. This working group will also aim to improve research communication internally within the nutrition science research community and foster improved interactions with stakeholders. This could work towards, for example, public–private partnership guidelines for effective and ethical collaborations to modify food products and food environments, allowing research and greater public interest to regain control of the steering wheel. This may also mean training or provision of tools for effective policy engagement, to enact change both as it relates to public health/environmental interventions as well as to research funding schemes. Together, these groups will devise productive ways to challenge the current structure of funding incentives that hinder systems-level or transdisciplinary approaches, to allow for the funding of more high-risk/high-gain interventions. With more than 4200 authors submitting research at the latest FENS gathering [40], and nearly 13,000 expected users of the FNH-RI (proposal in submission), organising around shared challenges and obstacles will surely generate the momentum needed to ensure nutrition sciences are fit for the twenty-first century.

## Conclusion

The grand challenge of meaningfully reducing malnutrition in all its forms, and halting the interrelated breakdown of the planetary systems that support all forms of life and health, can only be tackled by transdisciplinary approaches that consider the food system as a whole, including sociocultural factors, built off of collaborative platforms and consortia. Given that diet is the leading cause of poor health globally, and a major driver of the climate crisis, nutrition sciences have a key responsibility and a central role to play in addressing these challenges. Given its unique position at the intersection of multiple domains of science (Fig. 2), as well as at the intersection of both scientific and societal challenges, nutrition science is poised to form coherent initiatives and common policies to deliver meaningful and impactful research. This has so far been hindered by both organisational and funding issues, and the large corporate interest inherent in food that is largely concerned with profit and not collective public and environmental health. With improved communication and organisation, and together with societal groups, nutrition scientists can send a strong signal to industry, agriculture, retail and policymakers in demanding true health and environmental responsibility.

The various challenges related to what we eat have never been greater for humanity. The recently announced European Green New Deal [41] opens up significant resources for impactful research, but this comes with the burden of ensuring research communities and institutions are truly up to the task. For European nutrition scientists, this means re-evaluating the need for collaboration with other scientific domains to effectively tackle grand challenges. It also means the incorporation of innovative and transdisciplinary methods, as well as the training and facilities to do so. Such efforts will only succeed with a collective effort by the nutrition community to define the complex twenty-first-century challenges and concepts we are faced with, and to strategically and effectively communicate with both the public and policymakers. This will allow for truly impactful research, combatting malnutrition and consumer distrust, and promoting healthy and sustainable diets for all.
